# Visual Outcomes and Patient Satisfaction with Extended Monovision—An Innovative Strategy to Achieve Spectacle Independence in Refractive Lens Exchange

**DOI:** 10.3390/jcm14165684

**Published:** 2025-08-11

**Authors:** Dana Nagyova, Christoph Tappeiner, Andrej Blaha, David Goldblum, Dimitrios Kyroudis

**Affiliations:** 1 Department of Ophthalmology, Pallas Kliniken, 4600 Olten, Switzerlanddavid.goldblum@pallas-kliniken.ch (D.G.); dimitrios.kyroudis@pallas-kliniken.ch (D.K.); 2 Department of Ophthalmology, University Hospital Essen, University Duisburg-Essen, 45147 Essen, Germany; 3 Medical Faculty, University of Bern, 3008 Bern, Switzerland; 4 Major Health Economics and Healthcare Management, ZHAW Zurich University of Applied Sciences, 8400 Winterthur, Switzerland; 5 Medical Faculty, University of Basel, 4056 Basel, Switzerland

**Keywords:** extended monovision, refractive lens exchange (RLE), spectacle independence, patient satisfaction

## Abstract

**Background**: Spectacle independence is a key goal in refractive lens exchange (RLE), especially in younger, high-expectation patients. This study evaluates a novel extended monovision approach combining a monofocal aspheric intraocular lens (IOL) in the dominant eye with a rotationally asymmetric bifocal extended-depth-of-focus (EDOF) IOL in the non-dominant eye. The strategy aims to optimize full-range visual performance while minimizing photic phenomena. **Methods**: In this retrospective cohort study, presbyopic patients underwent bilateral RLE with a monofocal IOL (Hoya Vivinex XC1-SP; target: 0 diopters [D]) in the dominant eye and a rotationally asymmetric bifocal EDOF IOL (LENTIS LS-313 MF15; addition: +1.5 D; target: −1.25 D) in the non-dominant eye. Uncorrected distance visual acuity (UDVA, at 6 m), uncorrected intermediate visual acuity (UIVA, at 66 cm), and uncorrected near visual acuity (UNVA, at 36 cm) were assessed. Additional evaluations included binocular defocus curves, contrast sensitivity, stereoacuity, and photic phenomena. Spectacle independence and satisfaction were measured using the PRSIQ and NEI-RQL-42 questionnaires. **Results**: A total of 38 patients (76 eyes) were included. The mean postoperative binocular UDVA, UIVA, and UNVA were −0.03 ± 0.08, −0.08 ± 0.09, and 0.04 ± 0.08 logMAR, respectively. The defocus curve peaked at 0.0 D (6 m) with a mean visual acuity of −0.03 ± 0.08 logMAR. Functional vision better than 0.2 logMAR extended over defocus steps from +1.00 to −3.25 D. All patients were spectacle-independent for distance and intermediate vision, and 84% reported complete spectacle independence. Contrast sensitivity was within normal limits for age. Minimal photic phenomena were reported, and stereoacuity was preserved in 97% of patients (≤100 arcseconds). **Conclusions**: This innovative extended monovision approach, combining two different IOLs in a mini-monovision setup, provides excellent uncorrected visual acuity at all distances, high spectacle independence, and minimal side effects. It represents a compelling alternative to multifocal IOL implantation in presbyopic RLE candidates.

## 1. Introduction

Management strategies for presbyopia range from non-invasive options, such as spectacles and contact lenses, to surgical interventions, including corneal and scleral procedures, phakic intraocular lenses (IOLs), and refractive lens exchange (RLE) [1]. Patients opting for RLE are generally younger than typical cataract surgery candidates—often professionally active, in good general health, and with high expectations regarding spectacle independence and visual quality [2]. While optimal distance vision is a primary concern, these patients also value functional intermediate and near vision. As such, the selection of the most suitable IOLs requires meticulous preoperative assessment, patient counseling, and individualized planning [3].

Multifocal IOLs (MFIOLs) have been developed to offer a full range of vision without the need for spectacles [4]. However, their use is frequently associated with dysphotopsia and reduced performance in low-light conditions, which may lead to dissatisfaction or even IOL exchange [5]. In contrast, classical monovision—employing a refractive difference of 2 to 3 diopters between the eyes—can enable comfortable reading but is often limited by issues with binocular fusion and stereoacuity [6,7]. Moreover, monovision typically does not address intermediate vision effectively [8]. Mini-monovision (anisometropia between −0.75 D and −1.5 D), offers better tolerance and improved intermediate vision but still often necessitates reading glasses [9,10].

To overcome these limitations, we introduced the concept of extended monovision—using a low-addition bifocal extended-depth-of-focus (EDOF) IOL in the non-dominant eye with a monofocal IOL in the dominant eye [11]. The EDOF IOL’s rotationally asymmetric design reduces photic phenomena while maintaining depth of focus. The goal of extended monovision is to provide functional visual acuity across all distances while minimizing side effects.

This study investigates the visual and subjective outcomes of this approach in non-cataract patients undergoing RLE.

## 2. Patients and Methods

### 2.1. Study Design

This retrospective cohort study was conducted in accordance with the Declaration of Helsinki and approved by swissethics (No. 2022-02062; 15 March 2023). Informed consent for the use of clinical data was routinely obtained from all participants.

### 2.2. Patient Selection

Consecutive presbyopic patients undergoing bilateral sequential RLE were included. Inclusion criteria comprised a preoperative desire for spectacle independence, bilateral lens implantation, and a complete ophthalmological follow-up examination at least three months postoperatively. All patients underwent a preoperative monovision simulation with a low-addition multifocal contact lens in the non-dominant eye (target: −1.25 D) and a monofocal contact lens in the dominant eye (target: emmetropia). Exclusion criteria were strabismus with loss of stereoacuity, glaucoma with significant visual field damage or high progression risk, maculopathy, and corneal irregularities. Notably, previous corneal refractive surgery or preoperative astigmatism were not exclusion criteria.

### 2.3. Intraocular Lenses

The non-dominant eye received a bifocal extended-depth-of-focus (EDOF) IOL (LENTIS Comfort LS-313 MF15, Teleon Surgical B.V., Spankeren, The Netherlands) with a target spherical equivalent (SE) of −1.25 D. This plate-haptic, hydrophilic acrylic lens with hydrophobic surface properties features a rotationally asymmetric refractive design, combining an aspheric distance zone and a sectorial near zone (+1.5 D add). A smooth transition zone helps reduce light scatter [8]. The combination of IOL design and target refraction of −1.25 D should provide excellent intermediate visual acuity while also enabling functional near vision at approximately 36 cm (target refraction of −1.25 D and EDOF addition of +1.5 D results in an effective near addition of approximately +2.75 D).

The dominant eye was implanted with a monofocal aspheric IOL (Vivinex XC1-SP, Hoya Surgical Optics, Tokyo, Japan; target: 0 D), designed to minimize both spherical and coma aberrations through a negative aspheric balance curve [12].

IOL power was calculated using the Barrett Universal II formula based on measurements from either the IOL Master 700 (Carl Zeiss Meditec AG, Jena, Germany) or Lenstar LS900 (Haag-Streit, Köniz, Switzerland). For patients with prior refractive surgery, the ASCRS post-refractive calculator was employed. In cases of 0.75–1.0 D of corneal astigmatism, paired opposite clear corneal incisions were made; for higher astigmatism, toric IOLs were used.

### 2.4. Surgical Technique

All procedures were performed by a single experienced surgeon (D.K.) under topical anesthesia via a 2.4 mm clear corneal incision placed on the steepest meridian. Femtosecond laser-assisted RLE was offered using the VICTUS platform (Bausch & Lomb, Rochester, NY, USA) depending on patient preference. No intra- or postoperative complications occurred. The second eye was operated on within two weeks.

### 2.5. Examinations

All patients underwent a comprehensive preoperative ophthalmic assessment according to institutional standards. This included detailed medical history, slit-lamp and dilated fundus examination, subjective refraction, intraocular pressure measurement via Goldmann applanation tonometry, corneal topography using the Galilei G6 system (Ziemer Ophthalmic Systems AG, Port, Switzerland), and ocular biometry with either the IOLMaster 700 (Carl Zeiss Meditec AG, Jena, Germany) or Lenstar LS900 (Haag-Streit Group, Köniz, Switzerland). Ocular dominance was assessed with the hole-in-the-card test. Prior to surgery, a monovision simulation using contact lenses was performed to evaluate tolerance to the planned anisometropic setup.

Postoperative uncorrected distance visual acuity (UDVA, at 6 m), intermediate visual acuity (UIVA, at 66 cm), and near visual acuity (UNVA, at 36 cm) were measured monocularly and binocularly using the Jaeger chart under photopic conditions by the same examiner. Corrected distance visual acuity (CDVA) was determined after best spectacle correction.

Contrast sensitivity (CS) was measured binocularly at 6 m under mesopic conditions (3 cd/m^2^) using the ClearChart 4 system (Reichert Technologies, Depew, NY, USA). Sinusoidal gratings with vertical orientation were presented at spatial frequencies of 1.5, 3, 6, 12, and 18 cycles per degree (cpd). Log contrast sensitivity values were recorded and averaged.

Binocular defocus curves were assessed under uncorrected conditions using trial lenses ranging from +1.5 D to −3.5 D in randomized 0.25 D steps. Defocus ranges were defined as follows: near (−3.50 to −2.00 D, corresponding to 28–50 cm), intermediate (−2.00 to −0.50 D, 50 cm–2 m), and distance (−0.50 to +1.00 D, 2–6 m).

Stereoacuity at reading distance was evaluated using the Fly-S Stereo Acuity Test (Vision Assessment Corporation, Elk Grove Village, IL, USA) under both uncorrected and corrected conditions.

Photic phenomena were assessed using the Halo and Glare Simulator (Carl Zeiss Meditec AG, Jena, Germany), allowing patients to subjectively adjust halo and glare size and intensity on a scale from 0 to 100 to reflect their visual experience.

### 2.6. Patient-Reported Outcomes

Postoperative subjective visual function, spectacle independence, and overall satisfaction were assessed using two validated questionnaires. The Patient-Reported Spectacle Independence Questionnaire (PRSIQ) was used to evaluate the extent to which patients were able to perform daily visual tasks without the need for spectacles following lens implantation [13]. In addition, the National Eye Institute Refractive Error Quality of Life-42 (NEI-RQL-42) questionnaire was administered to assess the broader impact of refractive correction on patients’ daily life, visual clarity, and satisfaction [14,15]. This widely used instrument contains 42 items across 13 subscales and offers a detailed evaluation of vision-related quality of life.

## 3. Results

### 3.1. Patient Eligibility and Demographics

A total of 38 patients (76 eyes) were included in this retrospective analysis. The mean age was 62.3 ± 7.1 years (range, 44 to 75 years), and 17 participants (45%) were female. The mean axial length was 24.00 ± 1.85 mm, and the mean preoperative spherical equivalent (SE) was −0.20 ± 3.08 D. Preoperative CDVA averaged 0.03 ± 0.04 logMAR. All procedures were uneventful, with no intraoperative or postoperative complications. In total, 14 toric IOLs (18%) were implanted—7 monofocal and 7 EDOF. Femtosecond laser-assisted RLE was performed in seven patients.

### 3.2. Visual Outcomes

**Figure 1:**
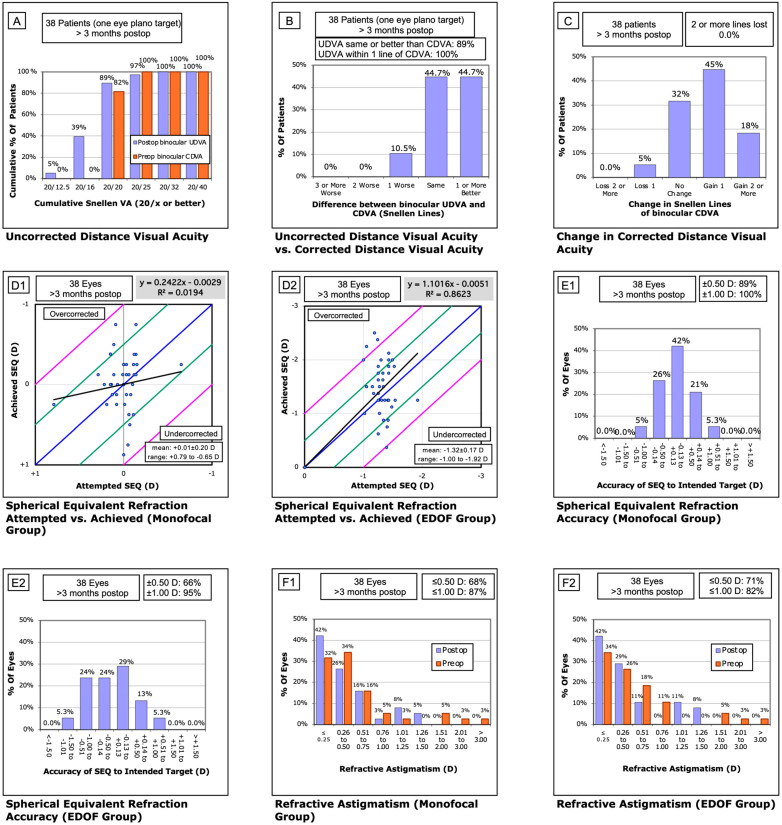
Standard refractive outcome graphs for the extended monovision cohort. (**A**) Binocular corrected distance visual acuity (CDVA) measured preoperatively and binocular uncorrected distance visual acuity (UDVA) measured >3 months postoperatively. (**B**) Change in binocular visual acuity from preoperative CDVA to postoperative UDVA. (**C**) Change in binocular CDVA from baseline to >3 months postoperatively. (**D1**,**D2**) Attempted versus achieved spherical equivalent in the monofocal (**D1**) and EDOF (**D2**) groups. (**E1**,**E2**) Refractive accuracy in the monofocal (**E1**) and EDOF (**E2**) groups >3 months postoperatively. (**F1**,**F2**) Residual astigmatism preoperatively and >3 months postoperatively in the monofocal (**F1**) and EDOF (**F2**) groups.

Right-eye dominance was observed in 30 patients (79%). Detailed visual and refractive results are presented in Table 1 and Figure 1. For eyes implanted with the monofocal IOL, the mean monocular UDVA (6 m), UIVA (66 cm), and UNVA (36 cm) were 0.00 ± 0.08, 0.16 ± 0.16, and 0.44 ± 0.15 logMAR, respectively. Eyes with the EDOF IOL achieved mean monocular UDVA, UIVA, and UNVA values of 0.38 ± 0.20, −0.05 ± 0.08, and 0.04 ± 0.08 logMAR, respectively. Mean binocular UDVA, UIVA, and UNVA were −0.03 ± 0.08, −0.08 ± 0.09, and 0.04 ± 0.08 logMAR, respectively. Overall, 90% of patients achieved a binocular UDVA of 0.00 logMAR or better.

The mean postoperative SE was −0.05 ± 0.35 D in dominant (monofocal) eyes and −1.51 ± 0.48 D in non-dominant (EDOF) eyes. Regression analysis of intended versus achieved SE demonstrated a slope of 0.24 for monofocal and 1.1 for EDOF lenses, with intercepts of 0.00 and R^2^ values of 0.02 and 0.86, respectively. The percentage of eyes within ±0.13 D of target refraction was 42% (*n* = 16) in dominant eyes and 29% (*n* = 11) in non-dominant eyes. Furthermore, 89% and 66% of eyes were within ±0.50 D, and 100% (*n* = 38) and 95% (*n* = 36) within ±1.00 D for the monofocal and EDOF groups, respectively. Residual astigmatism ≤0.25 D was observed in 42% of eyes in both groups, and ≤0.50 D in 68% and 71%, respectively.

### 3.3. Binocular Defocus Curve

**Figure 2:**
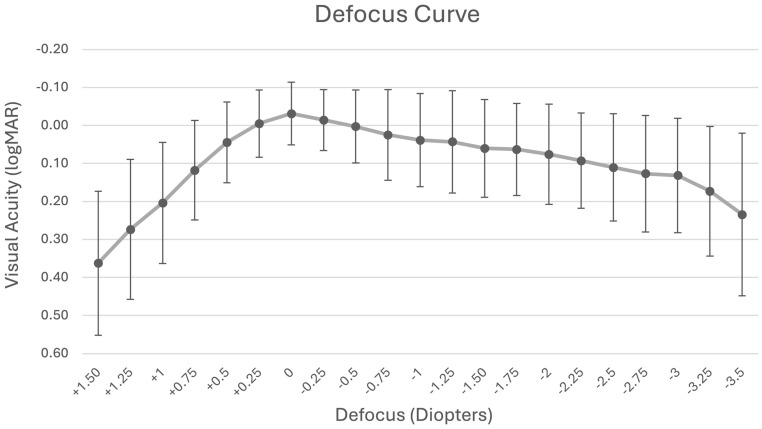
Mean binocular uncorrected defocus curve following refractive lens exchange with extended monovision.

The binocular defocus curve (Figure 2), assessed across a range of +1.50 D to −3.50 D in 0.25 D increments, showed peak visual acuity at 0.00 D (6 m) with a mean of −0.03 ± 0.08 logMAR. Visual acuity at −1.50 D (intermediate range) was 0.06 logMAR, while the values at −2.50 D and −3.00 D (near range) were 0.11 and 0.13 logMAR, respectively.

### 3.4. Stereoacuity

The mean uncorrected stereoacuity was 77.18 ± 74.61 arcseconds (1.74 ± 0.34 logMAR), and mean corrected stereoacuity was 41.21 ± 29.54 arcseconds (1.54 ± 0.24 logMAR). Good stereoacuity (≤100 arcseconds) was achieved by 30 patients (79%) without correction and by 37 patients (97%) with correction. One patient showed an uncorrected stereoacuity of 400 arcseconds due to an unknown cause. Excluding this case, the mean stereoacuity was 68.46 ± 53.15 arcseconds.

### 3.5. Contrast Sensitivity

**Figure 3:**
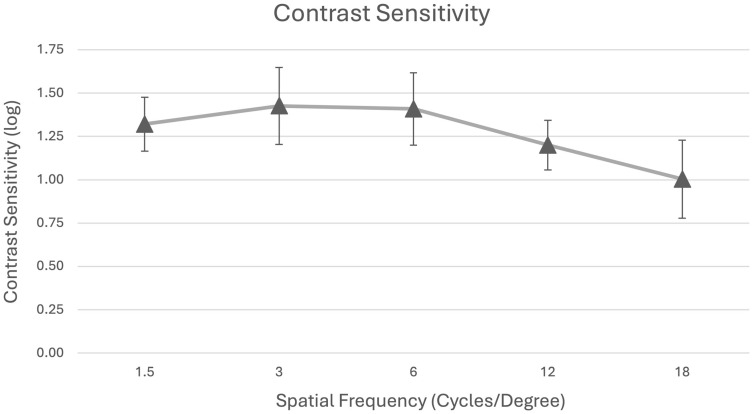
Mean binocular contrast sensitivity testing under mesopic conditions without glare.

Binocular contrast sensitivity under mesopic conditions without glare is summarized in Table 2 and Figure 3. The mean log contrast sensitivity values were 1.32 ± 0.15 (1.5 cpd), 1.43 ± 0.22 (3 cpd), 1.41 ± 0.21 (6 cpd), 1.20 ± 0.14 (12 cpd), and 1.00 ± 0.23 (18 cpd), remaining within the normal range for adults aged 50 years and older.

### 3.6. Halo and Glare Simulator

Halos were reported by 22 out of 38 patients (58%), with a mean perceived size of 34.77 ± 17.04 and a mean intensity score of 38.82 ± 13.21. Glare was reported by 17 patients (45%), with a mean size of 21.76 ± 14.26 and intensity of 36.94 ± 16.34.

### 3.7. Questionnaire

Of the 38 patients, 37 (97%) completed the postoperative questionnaires. According to the PRISQ, none of the respondents required spectacles for distance or intermediate vision, and 31 patients (84%) reported complete spectacle independence across all distances. Results from the NEI-RQL-42 questionnaire (Table 3) showed mean scores of 85.3 ± 25.9 for clarity of vision, 89.7 ± 17.4 for near vision, and 90.1 ± 18.6 for distance vision. The scores for glare, visual symptoms, and dependence on correction were 74.7 ± 27.1, 83.3 ± 25.1, and 93.9 ± 19.4, respectively.

## 4. Discussion

RLE patients typically expect not only spectacle independence, but also high-quality vision across all distances, including in challenging lighting conditions. In this context, extended monovision presents a promising alternative, potentially also for patients for whom a multifocal IOL is not ideally suitable or affordable. To our knowledge, this is the first study to report visual and patient-reported outcomes using an aspheric monofocal IOL in the dominant eye and a −1.25 D bifocal EDOF IOL in the non-dominant eye in a non-cataract RLE cohort. The concept leverages binocular summation and combines the optical clarity of a monofocal lens with the extended range provided by a low-addition EDOF IOL. Although this extended monovision approach induces anisometropia, patients tolerate it well, as binocular visual perception is primarily driven by the distance-dominant eye, with the monofocal IOL for far vision and the intermediate image formed by the EDOF eye. Our approach divides the range of focus between the two eyes, maintaining high contrast in the dominant eye through uncompromised monofocal optics, while providing a moderate range for intermediate and near distances. This minimizes photic phenomena due to the asymmetric refractive design of the EDOF IOL. A monofocal lens in the dominant eye provides the best possible vision and contrast without compromise, as compared to any alternative technology. EDOF technology offers good visual acuity, but it can still induce halos, glare, and reduced contrast at far distances compared to a monofocal lens, which we consider critical in any monovision setting [16,17,18,19,20], whereas the negative spherical aberration of a monofocal IOL reduces halos and optimizes contrast under mesopic conditions [21,22,23]. As observed in our results, this configuration enabled high-quality visual acuity at all distances, the preservation of stereopsis, and minimized photic phenomena—likely aided by the negative spherical aberration of the monofocal IOL and the refractive, rotationally asymmetric design of the EDOF lens. Our findings align with previous results in cataract patients treated with a similar strategy [11].

Compared to standard trifocal IOL implantation, our binocular UDVA, UIVA, and UNVA results compare favorably. Yim et al. reported values of 0.00 ± 0.04, 0.04, and 0.06 ± 0.17 logMAR for PanOptix lenses [3], while Khoramnia et al. showed slightly worse near performance with Clareon PanOptix (UNVA: 0.14 ± 0.10 logMAR) [24]. Similar outcomes were seen by Kretz et al. with AT Lisa tri 839MP implantation [25].

Our binocular defocus curve mirrors those of full-range IOLs classified by the ESCRS as having a continuous profile [26], with functional acuity maintained from +0.5 to −3.5 D. In comparison to PanOptix, our approach showed better acuity at vergences of 0 and −2.0 D, though PanOptix outperformed at −2.5 and −3.0 D [27]. Notably, our measurements were conducted under uncorrected, binocular conditions, enhancing their real-world relevance, whereas most comparator studies rely on best-corrected monocular testing.

As expected, contrast sensitivity was reduced under mesopic conditions at higher spatial frequencies. Nevertheless, performance remained within normative limits and exceeded that reported for bilateral AcrySof ReSTOR implantation using corrected testing conditions [28]. Additionally, our contrast sensitivity outcomes surpassed those reported for the Precizon Presbyopia IOL across all frequencies [29]. It is important to note that the available literature spans a wide range of models and generations, and direct comparisons should be interpreted cautiously. Especially, newer generations of trifocal IOLs, e.g., PanOptix (Alcon), AT Elana 841P (Zeiss), RayOne Galaxy (Rayner), FineVision HP (BVI Medical), enVista Envy (Bausch + Lomb Surgical), Tecnis Odyssey (Johnson & Johnson), and others, promise improved contrast sensitivity profiles compared to earlier models [30,31,32,33,34]. However, some of these recently introduced trifocal IOLs have not yet been evaluated in independent studies. Further research using standardized protocols across different IOL platforms is therefore needed to enable robust comparative assessments, particularly with respect to contrast sensitivity and visual phenomena.

Our uncorrected contrast sensitivity results are comparable to the published outcomes reported for multifocal IOLs, which often report contrast sensitivity values with best-correction and do not, therefore, account for potential optical degradation caused by residual refractive errors [35,36,37].

Stereoacuity was preserved in the majority of patients, with a mean value of 68.46 ± 53.15 arcseconds. This compares well with results from Hafez et al., who reported 79 ± 37 arcseconds in a mini-monovision setup [9]. These findings support the notion that mild anisometropia in extended monovision is well tolerated.

According to the questionnaire results, our approach shows comparable outcomes to the bilateral implantation of either trifocals or EDOF lenses [38,39,40,41].

In terms of spectacle independence, 84% of patients reported being fully free of glasses, and all were spectacle-free for distance and intermediate vision. These results are comparable to those reported by Akahoshi et al. with the FineVision HP IOL [42] and Fernández et al. using the AT LISA tri 839MP [43]. Our outcomes also exceeded those of Pedrotti et al. following implantation of the Lucidis IOL in all domains except glare [15]. Similarly, our results were superior to bilateral enhanced monofocal or EDOF combinations aimed at binocular emmetropia, particularly in the NEI-RQL-42 domains of spectacle dependence and symptoms [44].

Our study is limited by its retrospective design. This study represents an evaluation of our clinical experience with extended monovision. The absence of a direct control group, such as a parallel cohort with bilateral multifocal IOL implantation, limits our ability to make definitive conclusions about the comparative effectiveness of our approach. Future prospective, randomized studies with appropriate control arms are warranted to validate and generalize these preliminary observations. The results of binocular UDVA reached the same values as the defocus curve at distance. The defocus curve results for intermediate and near distances were slightly worse than those of UIVA and UNVA, which could potentially also be attributed to the increasing fatigue of the patients and/or ocular surface drying during prolonged examinations. Nevertheless, the results strongly support the clinical value of this extended monovision concept, particularly for discerning RLE candidates.

## 5. Conclusions

This study demonstrates that extended monovision—achieved by combining a monofocal aspheric IOL in the dominant eye with a low-add bifocal EDOF IOL in the non-dominant eye—can provide a level of spectacle independence comparable to bilateral multifocal IOL implantation. Extended monovision may represent a cost-effective alternative to trifocal IOLs, providing satisfactory visual performance across a wide range of distances. Importantly, this individualized approach offers additional advantages, such as reduced photic phenomena and high patient satisfaction. These findings support its suitability as a valuable alternative for visually demanding RLE patients.

## Figures and Tables

**Table 1:** Monocular and binocular uncorrected visual acuity (logMAR) for distance (UDVA, 6 m), intermediate (UIVA, 66 cm), and near (UNVA, 36 cm) vision following RLE with extended monovision. Data are presented as mean ± standard deviation (SD). IOL = intraocular lens; EDOF = extended-depth-of-focus.

	Eyes with Monofocal IOL (Mean ± SD)	Eyes with EDOF IOL (Mean ± SD)	Binocular (Mean ± SD)
UDVA (logMAR)	0.00 ± 0.08	0.38 ± 0.20	−0.03 ± 0.08
UIVA (logMAR)	0.16 ± 0.16	−0.05 ± 0.08	−0.08 ± 0.09
UNVA (logMAR)	0.44 ± 0.15	0.04 ± 0.08	0.04 ± 0.08

**Table 2:** Mean postoperative binocular contrast sensitivity under mesopic conditions across various spatial frequencies. All values were measured without refractive correction. C.S. = contrast sensitivity; log C.S. = logarithm of contrast sensitivity.

Spatial Frequency (cpd)	C.S.	Log C.S.
1.5	20.83 ± 10.88	1.32 ± 0.15
3	26.46 ± 16.44	1.43 ± 0.22
6	25.38 ± 14.90	1.41 ± 0.21
12	15.50 ± 6.40	1.20 ± 0.14
18	9.98 ± 7.64	1.00 ± 0.23

**Table 3:** Postoperative patient-reported outcomes from the NEI-RQL-42 questionnaire, assessing the impact of refractive correction on quality of life after refractive lens exchange (RLE). Values are presented as mean ± standard deviation (SD).

Measure	Mean ± SD
Clarity of vision	85.3 ± 25.9
Expectations	75.7 ± 37.9
Near vision	89.7 ± 17.4
Far vision	90.1 ± 18.6
Diurnal fluctuations	90.4 ± 18.9
Activity limitations	95.8 ± 15.5
Glare	74.7 ± 27.1
Symptoms	83.3 ± 25.1
Dependence on correction	93.9 ± 19.4
Worry	78.4 ± 23.4
Suboptimal correction	96.3 ± 14.6
Appearance	94.4 ± 19.4
Satisfaction with correction	90.3 ± 15.2

## Data Availability

The raw data supporting the conclusions of this article will be made available by the authors on request.
